# Efficacy of Dupilumab in the Treatment of Eosinophilic Esophagitis: A Systematic Review and Network Meta-Analysis of Randomized Controlled Trials

**DOI:** 10.3390/life15020307

**Published:** 2025-02-17

**Authors:** Szu-Hung Chu, Jeng-Jung Chen, Chung-Chu Chen, Wei-Te Lei, Chi-Hone Lien, Shung-Long Weng, Chun-Yan Yeung, Lawrence Yu-Ming Liu, Yu-Lin Tai, Ya-Ning Huang, Chien-Yu Lin

**Affiliations:** 1 Department of Pediatrics, Hsinchu Municipal MacKay Children’s Hospital, Hsinchu 30070, Taiwan; 4525@mmh.org.tw (S.-H.C.);; 2 Department of Biological Science and Technology, National Yang Ming Chiao Tung University, Hsinchu 30010, Taiwan; 3 Department of Internal Medicine, Hsinchu MacKay Memorial Hospital, Hsinchu 30071, Taiwan; 4 Centers of Natural Science, Minghsin University of Science and Technology, Hsinchu 30401, Taiwan; 5 Department of Medicine, MacKay Medical College, New Taipei 25245, Taiwan; 6 Graduate Institute of Clinical Medical Sciences, College of Medicine, Chang Gung University, Taoyuan 33302, Taiwan; 7 Department of Obstetrics and Gynecology, Hsinchu Municipal MacKay Children’s Hospital, Hsinchu 30070, Taiwan; 8 College of Public Health, National Taiwan University, Taipei 10055, Taiwan

**Keywords:** dupilumab, eosinophilic esophagitis, biological agents, monoclonal antibody

## Abstract

Eosinophilic esophagitis (EoE) is a chronic, immune-mediated disorder of the esophagus with rising prevalence. Dupilumab (DUPI), a monoclonal antibody that targets the interleukin-4 receptor α, has shown promise as a treatment option. We conducted a systematic review and network meta-analysis of randomized controlled trials searching the PubMed/Medline database, the Cochrane Database of Systematic Reviews, the Cochrane Central Register of Controlled Trials (CENTRAL), and the medRxiv preprint server up to 31 July 2024, assessing DUPI’s efficacy and optimal dosing in the treatment of EoE. Finally, three randomized-controlled trials comprising 470 participants, including 102 children under 12 years of age, were included in the qualitative synthesis. Both high-exposure (HE-DUPI, 300 mg weekly) and low-exposure (LE-DUPI, 300 mg biweekly) regimens achieved significant histologic remission relative to placebo (OR = 26.88, 95% CI 11.98–60.29 for LE-DUPI; OR = 29.15, 95% CI 13.68–62.12 for HE-DUPI). Although overall adverse events were comparable between groups, HE-DUPI was associated with a notable increase in serious adverse events. These findings suggest that DUPI is effective in promoting histologic remission in EoE, with LE-DUPI emerging as a preferred option for balancing efficacy and safety. This study highlights the efficacy and safety profiles of different dosing regimens and pediatric groups. Further studies are warranted to explore long-term outcomes and identify patient subgroups that may derive the greatest benefit from DUPI therapy.

## 1. Introduction

Eosinophilic esophagitis (EoE) is a chronic, immune-mediated esophageal disease characterized clinically by symptoms related to esophageal dysfunction and histologically by eosinophil-predominant inflammation [1]. Since its first description as a distinct clinicopathological entity in 1993, the understanding of EoE has evolved significantly [1,2]. The estimated prevalence of EoE ranges from 0.5 to 1 case per 1000 people, with an apparent increasing incidence over the past few decades [3,4,5]. Recent data indicate that the incidence is particularly high in developed countries, with rates as high as 5–10 new cases per 100,000 person-years [6].

The cardinal symptoms of EoE vary with age and include dysphagia, food impaction, chest pain, abdominal pain, and feeding disorders in younger children [7]. The disease course is typically chronic with fluctuating symptoms, significantly impacting patients’ quality of life through dietary restrictions, frequent medical visits, and psychological stress [8,9]. A strong association with atopic conditions has been observed, with studies reporting that 28 to 86% of adults and 42 to 93% of children with EoE have concurrent allergic diseases such as asthma, atopic dermatitis, or food allergies [10,11]. Recent genetic studies have identified several susceptibility loci, including variants in the TSLP/WDR36 and CAPN14 genes, suggesting a complex genetic architecture underlying the disease [12,13].

The diagnosis of EoE requires both clinical symptoms and histological findings, with a peak eosinophil count of ≥15 eosinophils per high-power field on esophageal biopsy a key diagnostic criterion [12,14]. Historically, dietary modifications and proton pump inhibitors have been the mainstay of treatment. However, the efficacy of these approaches is not always satisfactory, with studies showing response rates of 30–50% for proton pump inhibitors and varying success rates for dietary elimination strategies [8,15]. Given the underlying immune-mediated inflammatory nature of EoE, characterized by a type 2 inflammatory response with elevated levels of IL-4, IL-5, and IL-13, various immunomodulatory agents have been investigated as potential treatments [16]. Dupilumab (DUPI), a fully human monoclonal IgG4 antibody that blocks interleukin-4 receptor α and subsequent inflammatory cascades, has shown promise in treating several allergic diseases [17,18,19]. Its efficacy in reducing the clinical severity of conditions such as severe atopic dermatitis has been well documented, albeit with noted adverse effects including local reactions [20].

In May 2022, DUPI received approval from the US Food and Drug Administration for the treatment of EoE in adults and children older than 12 years [21]. This approval was based on phase 3 clinical trial data demonstrating significant improvements in both histological and clinical parameters [22]. Previous systematic reviews and meta-analyses of randomized controlled trials have demonstrated the clinical efficacy of DUPI in adults and adolescents [23]. Additionally, several real-world cohort studies have reported the promising efficacy of DUPI in the treatment of EoE, with no significant adverse events observed [24]. However, the efficacy and safety of DUPI in treating EoE in children under 12 years of age remain largely unclear. Moreover, following its approval, various dosing regimens have been implemented, yet the optimal therapeutic strategy remains to be determined. Furthermore, both randomized controlled trials (RCTs) and cohort studies are extensively utilized in clinical research, yet they differ significantly in study design, methodology, and application. RCTs are rigorously designed and carefully controlled to minimize confounding factors, thereby providing high-quality evidence on the efficacy and safety of investigational drugs [25]. To address these knowledge gaps, we conducted a systematic review and network meta-analysis of RCTs to comprehensively evaluate the clinical efficacy of DUPI in the treatment of EoE. By synthesizing data from randomized controlled trials, our study aims to provide an updated assessment of DUPI’s role in EoE management, with a particular focus on its use in young children, and to determine the optimal dosing regimen.

## 2. Materials and Methods

### 2.1. Study Design and Literature Search

This study was conducted in accordance with the Preferred Reporting Items for Systematic Reviews and Meta-Analyses (PRISMA) guidelines [26], which ensures transparent and complete reporting of systematic reviews and meta-analyses. Informed consent was waived due to the anonymous nature of the systematic review, as approved by our institutional review board. The ‘PICO’ (Patient, Intervention, Comparison, Outcome) criteria, a fundamental framework for evidence-based medicine research, were systematically applied for the literature search (Supplementary Table S1). The P refers to the patient population, including individuals with confirmed eosinophilic esophagitis (EoE), defined as ≥15 eosinophils per high-power field on esophageal biopsy. The intervention (I) was treatment with dupilumab (DUPI), a fully human monoclonal antibody targeting the IL-4/IL-13 pathway. The comparison group (C) consisted of individuals receiving a placebo or different dosing regimens of DUPI in randomized controlled trials. Finally, the outcomes measured (O) were histologic remission (defined as ≤6 eosinophils per high-power field) and treatment-emergent adverse events. Since the assessment of clinical EoE symptoms may be less reliable in young children, we utilized objective pathological findings as the primary outcome measure.

We conducted a comprehensive search of electronic medical databases using the primary terms ‘eosinophilic esophagitis’ and ‘dupilumab’, along with related keywords, Boolean operators (AND, OR, NOT), and Medical Subject Headings (MeSH) terms. The complete search strategy included variations of these terms such as ‘allergic esophagitis’, ‘anti-IL4 receptor alpha’, and ‘biologic therapy’. The databases searched included PubMed/Medline, the Cochrane Database of Systematic Reviews, the Cochrane Central Register of Controlled Trials (CENTRAL), and the medRxiv preprint server, with the search concluding on 31 July 2024. To maximize the capture of relevant studies, no restrictions were applied regarding language, publication year, or participant characteristics such as age, gender, or disease severity. Additionally, reference lists of included studies and relevant review articles were manually screened to identify any additional eligible studies. Two authors with expertise in systematic reviews conducted the literature search independently (JJ Chen and WT Lei) using standardized data extraction forms. Any disagreements or discrepancies in study selection or data extraction were resolved through discussion and consensus with a third author (SH Chu), who served as the arbitrator. The quality of included studies was assessed using the Cochrane risk of bias tool for randomized controlled trials.

### 2.2. Study Selection, Data Extraction, Systematic Review, and Meta-Analyses

We included randomized controlled trials (RCTs) that investigated the efficacy of DUPI in treating EoE. The exclusion criteria were as follows: duplicate publications from different databases, irrelevant articles (e.g., epidemiological studies or surveillance reports), studies that did not evaluate histologic remission or adverse events, case reports, animal studies, trials with no comparison arm, editorials, and review articles.

Different dosing regimens of DUPI for EoE were classified into high-exposure (HE-DUPI) and low-exposure (LE-DUPI) categories, based on study design. HE-DUPI referred to a weekly administration of 300 mg of DUPI, while LE-DUPI referred to a biweekly administration of 300 mg of DUPI for adults and adolescents. In children, HE-DUPI was designed to achieve steady-state trough concentrations similar to those from a weekly 300 mg administration in adults, while LE-DUPI corresponded to biweekly dosing [27].

The primary outcome was histologic remission of EoE, defined as a peak esophageal intraepithelial eosinophil count of ≤6 per high-power field (≤20 per square millimeter) at week 16. Secondary outcomes included adverse events reported by the participants. Two authors independently assessed and extracted data from the selected articles, including the first author’s name, study country, study type, participant population, age/gender, DUPI dosage, treatment interval, treatment duration, histologic outcomes, clinical outcomes, adverse events, and authors’ conclusions.

The quality of the included studies was assessed using the revised Cochrane risk-of-bias tool for randomized trials (RoB 2) [28]. Two authors independently evaluated study quality, focusing on domains such as selection, ascertainment, causality, and reporting. Any disagreements were resolved by discussion with a third author.

We conducted network meta-analyses using random-effects models to assess the efficacy of DUPI under different dosing schedules. Subgroup analysis and sensitivity tests were performed.

### 2.3. Statistical Analyses

Given the inherent variability in effect sizes across clinical studies, we employed a sophisticated random-effects regression model for meta-analyses, which allows for heterogeneity between study populations and methodological approaches. The Lu and Ades network meta-analysis model was implemented to comprehensively integrate both direct and indirect comparative evidence, enabling a more nuanced assessment of treatment effects across different study designs [29,30]. This approach provides a robust methodology for synthesizing complex clinical data while accounting for potential inter-study variations. Heterogeneity was rigorously assessed using the τ^2^ (tau-squared) statistic, a sophisticated measure that quantifies the variance in effect sizes between studies. The odds ratio (OR) was used to quantify the risk of incidence associated with exposure compared to placebo. To mitigate potential publication bias, a critical methodological concern in meta-analyses, we employed multiple complementary analytical techniques. Specifically, we utilized comparison-adjusted funnel plots, which offer a more refined visual representation of potential reporting biases compared to traditional funnel plot methods. Additionally, Egger’s test was conducted to statistically evaluate asymmetry in the research landscape, providing a quantitative assessment of potential publication bias.

To address potential inconsistencies within our network meta-analysis, we implemented two advanced methodological approaches: the loop-specific approach and node-splitting models. These sophisticated analytical techniques allow for a comprehensive evaluation of potential discrepancies between direct and indirect evidence within the research network. Statistical significance was conservatively defined as a *p*-value less than 0.05. All statistical analyses were performed using MedCalc (MedCalc Software, Ostend, Belgium) v18 and R software version 4.4.3 (R Foundation for Statistical Computing, Vienna, Austria), leveraging the advanced statistical capabilities of these robust computational platforms. This methodological approach ensures a comprehensive, rigorous, and transparent analysis of the available clinical evidence, providing a nuanced understanding of treatment effects while mitigating potential sources of bias and methodological heterogeneity.

## 3. Results

### 3.1. Study Selection Process

**Figure 1:**
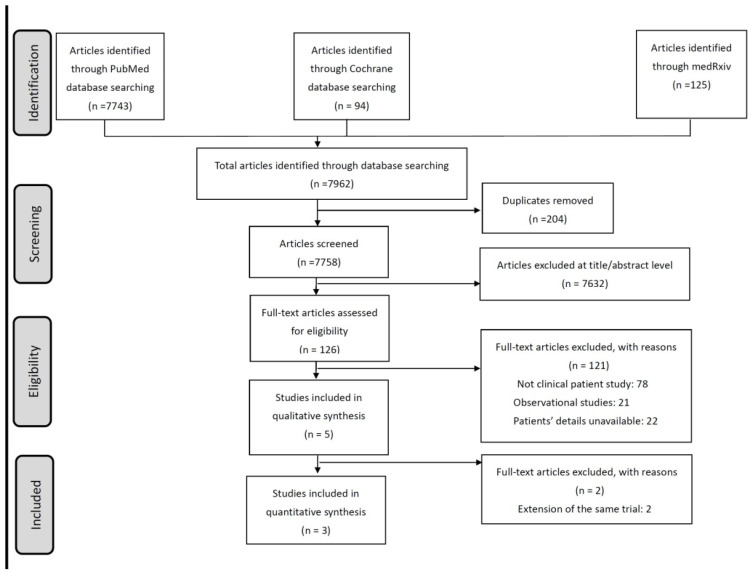
PRISMA flowchart of the literature search.

A comprehensive search of electronic databases up to 31 July 2024 was conducted to identify studies investigating dupilumab (DUPI) treatment for eosinophilic esophagitis (EoE) (Figure 1). Articles were excluded based on predetermined criteria, including duplicate publications, cohort studies, irrelevant studies, and review articles. Ultimately, five RCTs examining different DUPI dosing regimens were included in our analysis [22,27,31,32,33]. However, the publications by Rothenberg et al. and Bredenoord et al. were a follow-up study and a subgroup analysis study from a phase 3 trial, respectively, and were therefore excluded from the meta-analysis [22,32,33]. The overall methodological quality of these studies was high, with a low risk of bias (Supplementary Table S2). One study was a phase 2 trial published in 2020, while the other two were phase 3 trials (Table 1). Both phase 3 trials consisted of multiple parts, with some portions of the trials still ongoing. These trials were conducted across the United States, Australia, Canada, and Europe. A total of 470 participants, including 102 children under 12 years of age, were ultimately included in the qualitative synthesis.

### 3.2. Histologic Remission Outcomes

**Figure 2:**
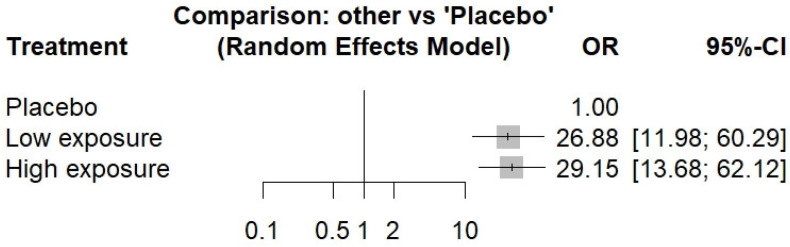
Forest plot of comparison of DUPI vs. placebo efficacy for histologic remission. OR, odds ratio; CI, confidence interval. The comparison presents the odds ratio for the incidence of histologic remission in low-exposure and high-exposure dupilumab groups versus placebo.

Histologic remission, reported consistently across all included studies, served as our primary outcome measure. A network meta-analysis was performed to compare treatment efficacy (network graph, Supplementary Figure S1). This network graph demonstrates direct comparisons among the HE-DUPI, LE-DUPI, and placebo treatments. The numbers on the edges indicate the number of studies supporting each comparison. The analysis revealed a significantly lower rate of histologic remission in the placebo group compared to the DUPI treatment groups (Supplementary Figure S2). Specifically, both HE-DUPI and LE-DUPI demonstrated significantly higher rates of histologic remission compared to placebo (Figure 2). HE-DUPI demonstrated a non-significantly higher remission rate compared to LE-DUPI (OR = 1.07, 95% CI 0.63–1.82). Sensitivity analyses were performed by sequentially removing individual trials to assess the robustness of our findings. While variations in the odds ratio were observed, the overall conclusion remained unchanged, confirming the reliability of our results. Subgroup analyses stratified by adults, adolescents, and young children yielded consistent results.

### 3.3. Adverse Events Analysis

**Figure 3:**
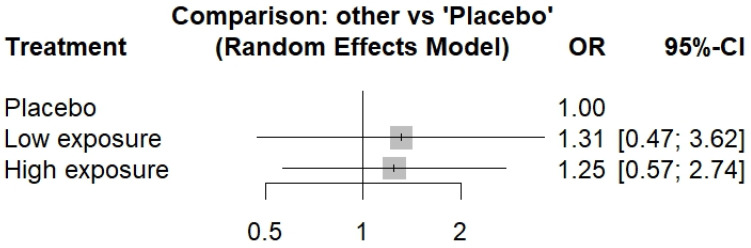
Forest plot of all adverse events in DUPI groups vs. placebo. OR, odds ratio; CI, confidence interval. The comparison presents the odds ratio for the incidence of all adverse events in the low-exposure and high-exposure dupilumab groups versus placebo.

**Figure 4:**
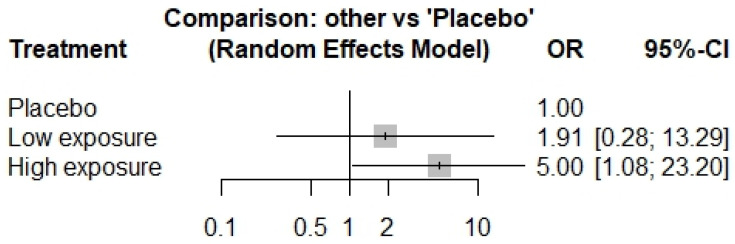
Forest plot of serious adverse events in DUPI groups vs. placebo. OR, odds ratio; CI, confidence interval. The comparison presents the odds ratio for the incidence of serious adverse events in low-exposure and high-exposure dupilumab groups versus placebo.

Patients receiving DUPI treatment exhibited a non-significant increase in the incidence of all adverse events compared to those receiving a placebo (Figure 3). Regarding serious adverse events (SAEs), a non-significant increased risk was observed in the LE-DUPI group (Figure 4). However, a statistically significant increase in SAEs was reported in patients receiving HE-DUPI.

## 4. Discussion

The management of EoE remains challenging for a subset of patients, and the recent approval of DUPI represents a novel therapeutic approach. Our meta-analysis of available RCTs demonstrates significant improvement in histologic remission among patients treated with both HE-DUPI and LE-DUPI dosing regimens. Additionally, our analysis included studies focusing on young children, showing efficacy and safety outcomes comparable to those observed in adult populations receiving DUPI. Regarding safety profiles, while HE-DUPI was associated with a higher incidence of SAEs, these were not attributed to the investigational product. Based on the current evidence, we propose that LE-DUPI may offer an optimal balance between efficacy and safety for the treatment of EoE.

Previous systematic reviews and meta-analyses have investigated the efficacy of DUPI in the treatment of EoE. Aziz et al. reviewed data from 287 patients across two RCTs and reported that DUPI demonstrated efficacy in both subjective and objective outcomes [23]. However, all the participants in their study were older than 12 years. In contrast, the present study includes 102 children younger than 12 years, thereby addressing the gap in the knowledge regarding the efficacy and safety of DUPI in the treatment of EoE in younger children. Additionally, Garg et al. conducted a systematic review and meta-analysis evaluating the real-world effectiveness of DUPI based on cohort studies [24]. While both RCTs and cohort studies are extensively utilized in clinical research, they differ significantly in study design, methodology, and application. RCTs are rigorously designed and carefully controlled to minimize confounding factors, thereby providing high-quality evidence on the efficacy and safety of investigational drugs [25]. The findings of the present study align with previous systematic reviews while supplementing critical data on the use of DUPI in children under 12 years of age.

DUPI, a human monoclonal antibody targeting the interleukin-4 receptor (IL-4R), inhibits downstream type 2 inflammatory cascades predominant in allergic diseases [17]. The strong association between EoE and atopic conditions, coupled with the elevated levels of IL-4, IL-13, and other inflammatory mediators observed in EoE patients, provides a robust theoretical framework for DUPI’s efficacy [34,35,36]. The drug’s mechanism of action, targeting the IL-4/IL-13 pathway, is particularly relevant to EoE pathophysiology, as these cytokines play crucial roles in tissue remodeling and eosinophil recruitment [37]. While dietary modifications and proton pump inhibitors remain first-line treatments and topical glucocorticoids such as budesonide and fluticasone are widely used, the introduction of DUPI expands the therapeutic armamentarium for clinicians [14,38]. Several cohort studies have explored the clinical efficacy of DUPI and demonstrated its promising effects in histologic improvement [24,39,40,41]. Improvements in clinical symptoms were also reported [24,40,42]. The present study, based on higher-level evidence from randomized controlled trials, reached the same conclusions as previous cohort studies.

The allergic etiology of EoE is further supported by its response to DUPI, an established treatment for other atopic conditions [36,43]. This therapeutic efficacy underscores the complex interplay between mucosal immunity, allergen exposure, and esophageal inflammation in EoE pathogenesis. The benefits of DUPI for EoE in adults and adolescents are relatively well established; however, its efficacy in children remains largely unclear [44]. A study by Chehade et al. provided compelling evidence supporting the histologic efficacy of DUPI in treating pediatric EoE [27]. Previous systematic reviews and meta-analyses examined the efficacy of DUPI in real-world cohort studies [24,41]. However, the efficacy of DUPI in young children was not addressed in these reviews. Our study strengthens the evidence of DUPI’s efficacy specifically in the pediatric population, particularly young children. Further sensitivity analyses and subgroup analyses stratified by adults, adolescents, and young children yielded consistent results. DUPI has been used for several years to manage severe atopic dermatitis in children, with well-documented efficacy and safety profiles. Transient increases in eosinophil count have been observed in children undergoing DUPI treatment for allergic diseases; however, this elevation did not compromise clinical efficacy [45]. DUPI’s application in the treatment of refractory EoE in children thus represents a reasonable therapeutic option for pediatricians [46,47].

The severity of EoE is primarily assessed using clinical symptoms, endoscopic findings, and histologic pathology [6,8]. Several scoring systems have been developed, and the correlation between clinical symptoms and pathological changes has been well established [48]. Our review focuses on children with EoE, where subjective symptom evaluation may be less reliable, particularly in younger children. Consequently, the primary outcome of our study emphasizes objective histologic remission. Additionally, the long-term efficacy of DUPI treatment remains largely uncertain, although previous studies have documented its 1-year efficacy in treating atopic dermatitis and EoE [20,32]. Future research investigating the long-term efficacy of DUPI is highly warranted.

Adverse events, particularly SAEs, are a critical consideration in clinical trials. Although the safety of DUPI has been well documented and no safety concerns were observed in a previous study [49], across the three trials analyzed, twenty-two participants experienced SAEs: fifteen in the HE-DUPI group, six in the LE-DUPI group, and one in the placebo group. The reported SAEs encompassed a diverse range of conditions, including procedural complications, infectious events, psychiatric manifestations, and systemic reactions. Notably, all the SAEs were deemed unrelated to the investigational product by the trial investigators. Non-serious adverse events occurred in approximately 75% of participants across all groups, with common manifestations including COVID-19, injection site reactions, upper respiratory tract infections, and various systemic symptoms. Our analysis revealed a non-significant increase in the overall number of AEs in both DUPI groups, but a significant increase in the number of SAEs in the HE-DUPI group. This safety profile informs our recommendation of LE-DUPI as the preferred regimen in patients with refractory EoE.

The strengths of our review lie in the inclusion of well-designed RCTs with robust methodological quality. Young children undergoing DUPI treatment were also included in our review. Additionally, we compared the histologic efficacy and adverse event profiles of different DUPI dosing regimens through a network meta-analysis. However, several limitations warrant consideration. Firstly, the absence of pharmacokinetic data on DUPI exposure in individual participants precludes a detailed analysis of dose–response relationships and potential correlations with adverse events. Secondly, while we focused on histologic remission as the primary outcome, the assessment of patient-reported outcomes and their correlation with histologic findings would provide a more comprehensive understanding of DUPI’s clinical impact. Assessing subjective symptoms in young children remains challenging. Third, although the enrolled RCTs were of high quality and demonstrated a low risk of bias, several confounding factors may have influenced the clinical efficacy observed, such as differences in inclusion criteria, variations in disease severity, and prior treatment histories within the study populations. Finally, further studies are needed to explore the long-term efficacy of DUPI in treating EoE [50,51].

## 5. Conclusions

Eosinophilic esophagitis is a challenging condition with a clear immunological basis. Our meta-analysis demonstrates significant improvement in histologic remission with DUPI treatment. While HE-DUPI showed a higher incidence of SAEs, these were not attributed to the treatment itself. Based on the current evidence, we recommend LE-DUPI as a promising therapeutic option for patients with EoE who have shown inadequate response to conventional treatments. Further research is warranted to elucidate long-term efficacy, optimize dosing regimens, and identify patient subgroups most likely to benefit from this targeted biological therapy.

## Figures and Tables

**Table 1:** Characteristics of enrolled trials.

Study	Country	Part	Participants Number, Total (Each Group)	Age in Each Group (Years Old)	Gender in Each Group (M/F)	Intervention and Comparison Groups		Outcomes Extraction	Selected in Final Meta-Analysis
						Dose and Frequency	Duration		
Hirano 2020 [31]	United States		47 (23 + 24)	33.1, 36.1	13/10, 10/14	300 mg weekly vs. placebo	12 weeks	Change in SDI PRO score Histologic measures of type 2 inflammation, endoscopically visualized anatomic measures, distensibility measures of esophagus Adverse events	Yes
Dellon 2022 [22]	Australia, Canada, Europe, United States	A	81 (42 + 39)	33.9, 28.8	28/14, 21/18	300 mg weekly vs. placebo	24 weeks	Histologic remission The absolute change from baseline in the DSQ score Other secondary outcomes Adverse events	Yes
		B	240 (80 + 81 + 79)	28.7, 27.8, 27.9	50/30, 45/36, 58/21	300 mg weekly vs. 300 mg biweekly vs. placebo	24 weeks		Yes
		A → C	40 + 37			300 mg weekly → 300 mg weekly vs. placebo → 300 mg weekly	28 weeks		No
		B → C	Ongoing						No
Chehade 2024 [27]	United States, Canada	A	102 (37 + 31 + 34)	6.8, 7.2, 7.2	28/9, 25/6, 25/9	HE-DUPI vs. LE-DUPI vs. placebo ^#^	16 weeks	Histologic remission Secondary histologic and clinical endpoints Adverse events	Yes
		B	Ongoing, following part A				36 weeks		No
		C	Ongoing				108 weeks		No

Abbreviations: DSQ, Dysphagia Symptom Questionnaire; HE-DUPI, high-exposure dupilumab; LE-DUPI, low-exposure dupilumab; SDI, Straumann Dysphagia Instrument. # The trough level at a steady state of HE-DUPI is equal to 300 mg weekly administration in adults and that of LE-DUPI is equal to 300 mg biweekly administration, respectively.

## Supplementary Materials

The following supporting information can be downloaded at: https://www.mdpi.com/article/10.3390/life15020307/s1, Figure S1: Netgraph of enrolled studies; Figure S2: Forest plot of DUPI efficacy for histologic remission, Table S1: PICO of present systematic review. Table S2: RoB 2 bias risk assessment of enrolled trials.

## References

[1] Liacouras C.A., Furuta G.T., Hirano I., Atkins D., Attwood S.E., Bonis P.A., Burks A.W., Chehade M., Collins M.H., Dellon E.S. (2011). Eosinophilic esophagitis: Updated consensus recommendations for children and adults. J. Allergy Clin. Immunol..

[2] Dellon E.S., Hirano I. (2018). Epidemiology and natural history of eosinophilic esophagitis. Gastroenterology.

[3] Kapel R.C., Miller J.K., Torres C., Aksoy S., Lash R., Katzka D.A. (2008). Eosinophilic esophagitis: A prevalent disease in the united states that affects all age groups. Gastroenterology.

[4] Arias Á., Pérez-Martínez I., Tenías J.M., Lucendo A.J. (2016). Systematic review with meta-analysis: The incidence and prevalence of eosinophilic oesophagitis in children and adults in population-based studies. Aliment. Pharmacol. Ther..

[5] Spergel J.M., Book W.M., Mays E., Song L., Shah S.S., Talley N.J., Bonis P.A. (2011). Variation in prevalence, diagnostic criteria, and initial management options for eosinophilic gastrointestinal diseases in the united states. J. Pediatr. Gastroenterol. Nutr..

[6] Muir A., Falk G.W. (2021). Eosinophilic esophagitis: A review. JAMA.

[7] Furuta G.T., Katzka D.A. (2015). Eosinophilic esophagitis. N. Engl. J. Med..

[8] Lucendo A.J., Molina-Infante J., Arias Á., von Arnim U., Bredenoord A.J., Bussmann C., Amil Dias J., Bove M., González-Cervera J., Larsson H. (2017). Guidelines on eosinophilic esophagitis: Evidence-based statements and recommendations for diagnosis and management in children and adults. United Eur. Gastroenterol. J..

[9] Taft T.H., Kern E., Keefer L., Burstein D., Hirano I. (2011). Qualitative assessment of patient-reported outcomes in adults with eosinophilic esophagitis. J. Clin. Gastroenterol..

[10] Simon D., Cianferoni A., Spergel J.M., Aceves S., Holbreich M., Venter C., Rothenberg M.E., Terreehorst I., Muraro A., Lucendo A.J. (2016). Eosinophilic esophagitis is characterized by a non-ige-mediated food hypersensitivity. Allergy.

[11] Spergel J.M., Brown-Whitehorn T.F., Beausoleil J.L., Franciosi J., Shuker M., Verma R., Liacouras C.A. (2009). 14 years of eosinophilic esophagitis: Clinical features and prognosis. J. Pediatr. Gastroenterol. Nutr..

[12] Hirano I., Moy N., Heckman M.G., Thomas C.S., Gonsalves N., Achem S.R. (2013). Endoscopic assessment of the oesophageal features of eosinophilic oesophagitis: Validation of a novel classification and grading system. Gut.

[13] Kottyan L.C., Davis B.P., Sherrill J.D., Liu K., Rochman M., Kaufman K., Weirauch M.T., Vaughn S., Lazaro S., Rupert A.M. (2014). Genome-wide association analysis of eosinophilic esophagitis provides insight into the tissue specificity of this allergic disease. Nat. Genet..

[14] Dellon E.S., Gonsalves N., Hirano I., Furuta G.T., Liacouras C.A., Katzka D.A. (2013). ACG clinical guideline: Evidenced based approach to the diagnosis and management of esophageal eosinophilia and eosinophilic esophagitis (EOE). Am. J. Gastroenterol..

[15] Hahn J.W., Lee K., Shin J.I., Cho S.H., Turner S., Shin J.U., Yeniova A., Koyanagi A., Jacob L., Smith L. (2023). Global incidence and prevalence of eosinophilic esophagitis, 1976–2022: A systematic review and meta-analysis. Clin. Gastroenterol. Hepatol..

[16] Eluri S., Selitsky S.R., Perjar I., Hollyfield J., Betancourt R., Randall C., Rusin S., Woosley J.T., Shaheen N.J., Dellon E.S. (2019). Clinical and molecular factors associated with histologic response to topical steroid treatment in patients with eosinophilic esophagitis. Clin. Gastroenterol. Hepatol..

[17] Beck L.A., Thaçi D., Hamilton J.D., Graham N.M., Bieber T., Rocklin R., Ming J.E., Ren H., Kao R., Simpson E. (2014). Dupilumab treatment in adults with moderate-to-severe atopic dermatitis. N. Engl. J. Med..

[18] Ledda A.G., Costanzo G., Sambugaro G., Caruso C., Bullita M., Di Martino M.L., Serra P., Firinu D., Del Giacco S. (2023). Eosinophil cationic protein variation in patients with asthma and crswnp treated with dupilumab. Life.

[19] Marron S.E., Tomas-Aragones L., Moncin-Torres C.A., Gomez-Barrera M., Aranibar F.J.G.-L.d. (2021). Patient reported outcome measure in atopic dermatitis patients treated with dupilumab: 52-weeks results. Life.

[20] Blauvelt A., de Bruin-Weller M., Gooderham M., Cather J.C., Weisman J., Pariser D., Simpson E.L., Papp K.A., Hong H.C., Rubel D. (2017). Long-term management of moderate-to-severe atopic dermatitis with dupilumab and concomitant topical corticosteroids (liberty ad chronos): A 1-year, randomised, double-blinded, placebo-controlled, phase 3 trial. Lancet.

[21] U.S. Food and Drug Administration FDA Approves First Treatment for Eosinophilic Esophagitis, a Chronic Immune Disorder. FDA News Release. https://www.prnewswire.com/news-releases/fda-approves-first-treatment-for-eosinophilic-esophagitis-a-chronic-immune-disorder-301552266.html.

[22] Dellon E.S., Rothenberg M.E., Collins M.H., Hirano I., Chehade M., Bredenoord A.J., Lucendo A.J., Spergel J.M., Aceves S., Sun X. (2022). Dupilumab in adults and adolescents with eosinophilic esophagitis. N. Engl. J. Med..

[23] Aziz M., Haghbin H., Gangwani M., Aziz A., Dahiya D.S., Ali H., Lee-Smith W., Goyal H., Kamal F. (2024). Efficacy of dupilumab in eosinophilic esophagitis: A systematic review and meta-analysis of randomized controlled trials. Am. J. Ther..

[24] Garg A., Moond V., Broder A., Mohan B. (2024). S716 real-world effectiveness of dupilumab in eosinophilic esophagitis: A systematic review and meta-analysis. Off. J. Am. Coll. Gastroenterol. ACG.

[25] Hong Y.D., Jansen J.P., Guerino J., Berger M.L., Crown W., Goettsch W.G., Mullins C.D., Willke R.J., Orsini L.S. (2021). Comparative effectiveness and safety of pharmaceuticals assessed in observational studies compared with randomized controlled trials. BMC Med..

[26] Hutton B., Salanti G., Caldwell D.M., Chaimani A., Schmid C.H., Cameron C., Ioannidis J.P., Straus S., Thorlund K., Jansen J.P. (2015). The prisma extension statement for reporting of systematic reviews incorporating network meta-analyses of health care interventions: Checklist and explanations. Ann. Intern. Med..

[27] Chehade M., Dellon E.S., Spergel J.M., Collins M.H., Rothenberg M.E., Pesek R.D., Hirano I., Liu R., Laws E., Mortensen E. (2024). Dupilumab for eosinophilic esophagitis in patients 1 to 11 years of age. N. Engl. J. Med..

[28] Sterne J.A.C., Savović J., Page M.J., Elbers R.G., Blencowe N.S., Boutron I., Cates C.J., Cheng H.-Y., Corbett M.S., Eldridge S.M. (2019). Rob 2: A revised tool for assessing risk of bias in randomised trials. BMJ.

[29] Lu G., Ades A.E. (2004). Combination of direct and indirect evidence in mixed treatment comparisons. Stat. Med..

[30] Chiu N.C., Chi H., Tu Y.K., Huang Y.N., Tai Y.L., Weng S.L., Chang L., Huang D.T., Huang F.Y., Lin C.Y. (2021). To mix or not to mix? A rapid systematic review of heterologous prime-boost COVID-19 vaccination. Expert. Rev. Vaccines.

[31] Hirano I., Dellon E.S., Hamilton J.D., Collins M.H., Peterson K., Chehade M., Schoepfer A.M., Safroneeva E., Rothenberg M.E., Falk G.W. (2020). Efficacy of dupilumab in a phase 2 randomized trial of adults with active eosinophilic esophagitis. Gastroenterology.

[32] Rothenberg M.E., Dellon E.S., Collins M.H., Hirano I., Chehade M., Bredenoord A.J., Lucendo A.J., Spergel J.M., Sun X., Hamilton J.D. (2023). Efficacy and safety of dupilumab up to 52 weeks in adults and adolescents with eosinophilic oesophagitis (liberty eoe treet study): A multicentre, double-blind, randomised, placebo-controlled, phase 3 trial. Lancet Gastroenterol. Hepatol..

[33] Bredenoord A.J., Dellon E.S., Hirano I., Lucendo A.J., Schlag C., Sun X., Glotfelty L., Mannent L., Maloney J., Laws E. (2024). Dupilumab demonstrated efficacy and was well tolerated regardless of prior use of swallowed topical corticosteroids in adolescent and adult patients with eosinophilic oesophagitis: A subgroup analysis of the phase 3 liberty eoe treet study. Gut.

[34] Chehade M., Falk G.W., Aceves S., Lee J.K., Mehta V., Leung J., Shumel B., Jacob-Nara J.A., Deniz Y., Rowe P.J. (2022). Examining the role of type 2 inflammation in eosinophilic esophagitis. Gastro Hep Adv..

[35] Syverson E.P., Rubinstein E., Lee J.J., McDonald D.R., Hait E. (2024). The role of dupilumab in the treatment of eosinophilic esophagitis. Immunotherapy.

[36] Lugović-Mihić L., Meštrović-Štefekov J., Potočnjak I., Cindrić T., Ilić I., Lovrić I., Skalicki L., Bešlić I., Pondeljak N. (2023). Atopic dermatitis: Disease features, therapeutic options, and a multidisciplinary approach. Life.

[37] Gandhi N.A., Pirozzi G., Graham N.M.H. (2017). Commonality of the il-4/il-13 pathway in atopic diseases. Expert. Rev. Clin. Immunol..

[38] Chan J., Flynn D.M., Gordon M., Parmar R., Moolenschot K., Jackman L., Gaynor E., Epstein J., Cordell A., Kannappan H. (2024). Swallowed topical steroid therapy for eosinophilic oesophagitis in children: Practical, evidence-based guidance by the bspghan eosinophilic oesophagitis working group. BMJ Paediatr. Open.

[39] Becker R., Rigsby M., Suchi M., Lerner D.G., Chugh A. (2024). Dupilumab in adolescent eosinophilic esophagitis: Experience with fibrostenosis and eosinophilic gastrointestinal disease with esophageal involvement. J. Pediatr. Gastroenterol. Nutr..

[40] Lee C.J., Dellon E.S. (2024). Real-world efficacy of dupilumab in severe, treatment-refractory, and fibrostenotic patients with eosinophilic esophagitis. Clin. Gastroenterol. Hepatol..

[41] Russin M., Chen J., Rubenstein J.H., Chang J.W. (2024). Real-world effectiveness and use of dupilumab in eosinophilic esophagitis. Am. J. Gastroenterol..

[42] Spergel J.M., Chehade M., Dellon E.S., Bredenoord A.J., Sun X., Glotfelty L., Shabbir A., Tilton S.T., McCann E. (2024). Dupilumab improves health-related quality of life and a range of symptoms in patients with eosinophilic esophagitis. Am. J. Gastroenterol..

[43] Aceves S.S., Dellon E.S., Greenhawt M., Hirano I., Liacouras C.A., Spergel J.M. (2023). Clinical guidance for the use of dupilumab in eosinophilic esophagitis: A yardstick. Ann. Allergy Asthma Immunol..

[44] de Oliveira F.D., Costa R.C., de Santana Sato E.D.B., Khalil S.M., Meine G.C. (2024). Efficacy and safety of monoclonal antibodies for the treatment of eosinophilic esophagitis: A systematic review and meta-analysis of randomized controlled trials. Dig. Dis. Sci..

[45] Wechsler M.E., Klion A.D., Paggiaro P., Nair P., Staumont-Salle D., Radwan A., Johnson R.R., Kapoor U., Khokhar F.A., Daizadeh N. (2022). Effect of dupilumab on blood eosinophil counts in patients with asthma, chronic rhinosinusitis with nasal polyps, atopic dermatitis, or eosinophilic esophagitis. J. Allergy Clin. Immunol. Pract..

[46] Caminati M., Senna G., Maule M., Di Sabatino A., Rossi C.M. (2024). Diagnosis, management and therapeutic options for eosinophilic esophagitis. Curr. Opin. Allergy Clin. Immunol..

[47] Chawla K., Alabbas B., Sheth D., Papademetriou M. (2020). As easy as eoe: A novel and effective multidisciplinary approach to care of patients with eosinophilic esophagitis in the age of biologics. Dig. Dis. Sci..

[48] Dellon E.S., Khoury P., Muir A.B., Liacouras C.A., Safroneeva E., Atkins D., Collins M.H., Gonsalves N., Falk G.W., Spergel J.M. (2022). A clinical severity index for eosinophilic esophagitis: Development, consensus, and future directions. J. Allergy Clin. Immunol..

[49] Sambugaro G., Brambilla E., Costanzo G., Bonato V., Ledda A.G., Del Giacco S., Scarpa R., Rattazzi M., Favero E., Cinetto F. (2024). COVID-19 clinical features and outcome in italian patients treated with biological drugs targeting type 2 inflammation. Life.

[50] González-Uribe V., Rodríguez-Bueno C.P.A., Mojica-González Z.S., Malagón-Liceaga A., Basile-Alvarez M.R. (2024). Sustained clinical and histopathological remission in a patient with eosinophilic esophagitis and type-2 comorbidities at 18 months after discontinuation of dupilumab. Clin. J. Gastroenterol..

[51] Rothenberg M., Dellon E., Bredenoord A., Collins M., Hirano I., Chehade M., Lucendo A., Spergel J., Sun X., Hamilton J. (2022). Dupilumab improves clinical and histologic aspects of disease in adult and adolescent patients with eosinophilic esophagitis at week 24: Results from part b of the 3-part liberty eoe treet study. J. Allergy Clin. Immunol..

