# Supplementary material for: Efficacy of Dupilumab in the Treatment of Eosinophilic Esophagitis: A Systematic Review and Network Meta-Analysis of Randomized Controlled Trials
Source: Life (Basel). 2025 Feb 17;15(2):307. doi: 10.3390/life15020307 (PMC11857325; doi:10.3390/life15020307)
Supplement: Supplementary file 1 [file life-15-00307-s001.zip › Supplementary Table S1 new.pdf]

Supplementary Table S1 PICO of present systematic review

P	I	C	O
Patient	Intervention	Comparison	Outcomes
Patients with confirmed eosinophilic esophagitis	Treatment with dupilumab (DUPI)	Individuals receiving placebo or different dosing regimens of DUPI in randomized controlled trials.	Histologic remission (defined as ≤6 eosinophils per high-power field) and treatment-emergent adverse events
