# Supplementary material for: Efficacy of Dupilumab in the Treatment of Eosinophilic Esophagitis: A Systematic Review and Network Meta-Analysis of Randomized Controlled Trials
Source: Life (Basel). 2025 Feb 17;15(2):307. doi: 10.3390/life15020307 (PMC11857325; doi:10.3390/life15020307)
Supplement: Supplementary file 1 [file life-15-00307-s001.zip › Supplementary Table S2_Rob2 risk assessment.pdf]

Supplementary Table S2. RoB 2 bias risk assessment of enrolled trials

Bias domain	Randomization	Deviations from Intended Interventions	Missing Outcome Data	Bias in Measurement of the Outcome	Bias in Selection of the Reported Result	Overall bias
Hirano 2020	Low	Low	Low	Low	Low	Low
Dellon 2022	Low	Low	Low	Low	Low	Low
Chehade 2024	Low	Low	Low	Low	Low	Low
