# Supplementary figures and images for: Efficacy of Dupilumab in the Treatment of Eosinophilic Esophagitis: A Systematic Review and Network Meta-Analysis of Randomized Controlled Trials
Source: Life (Basel). 2025 Feb 17;15(2):307. doi: 10.3390/life15020307 (PMC11857325; doi:10.3390/life15020307)

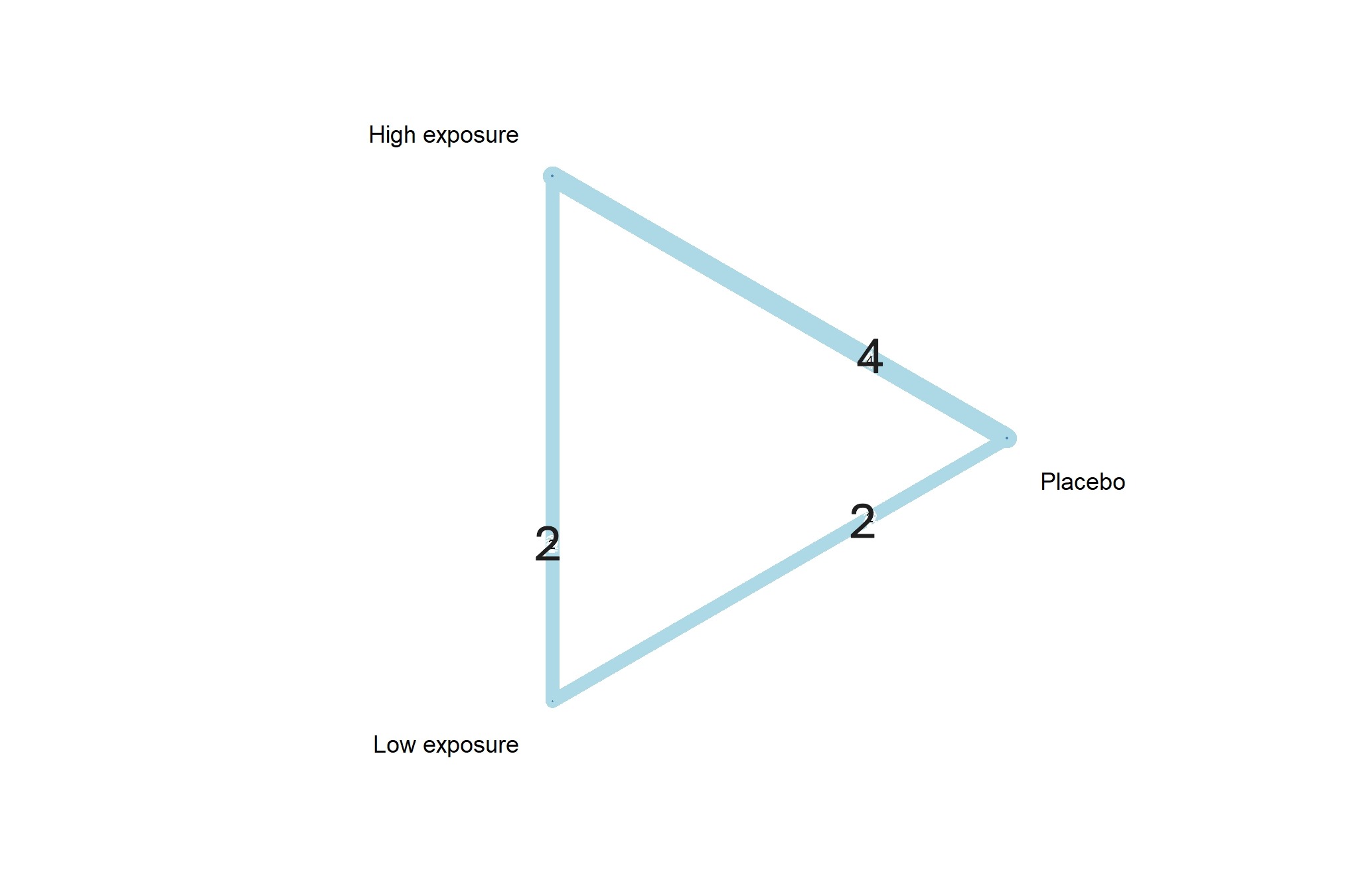

Supplement: Supplementary file 1 [file life-15-00307-s001.zip › Figure S1 new.JPG]

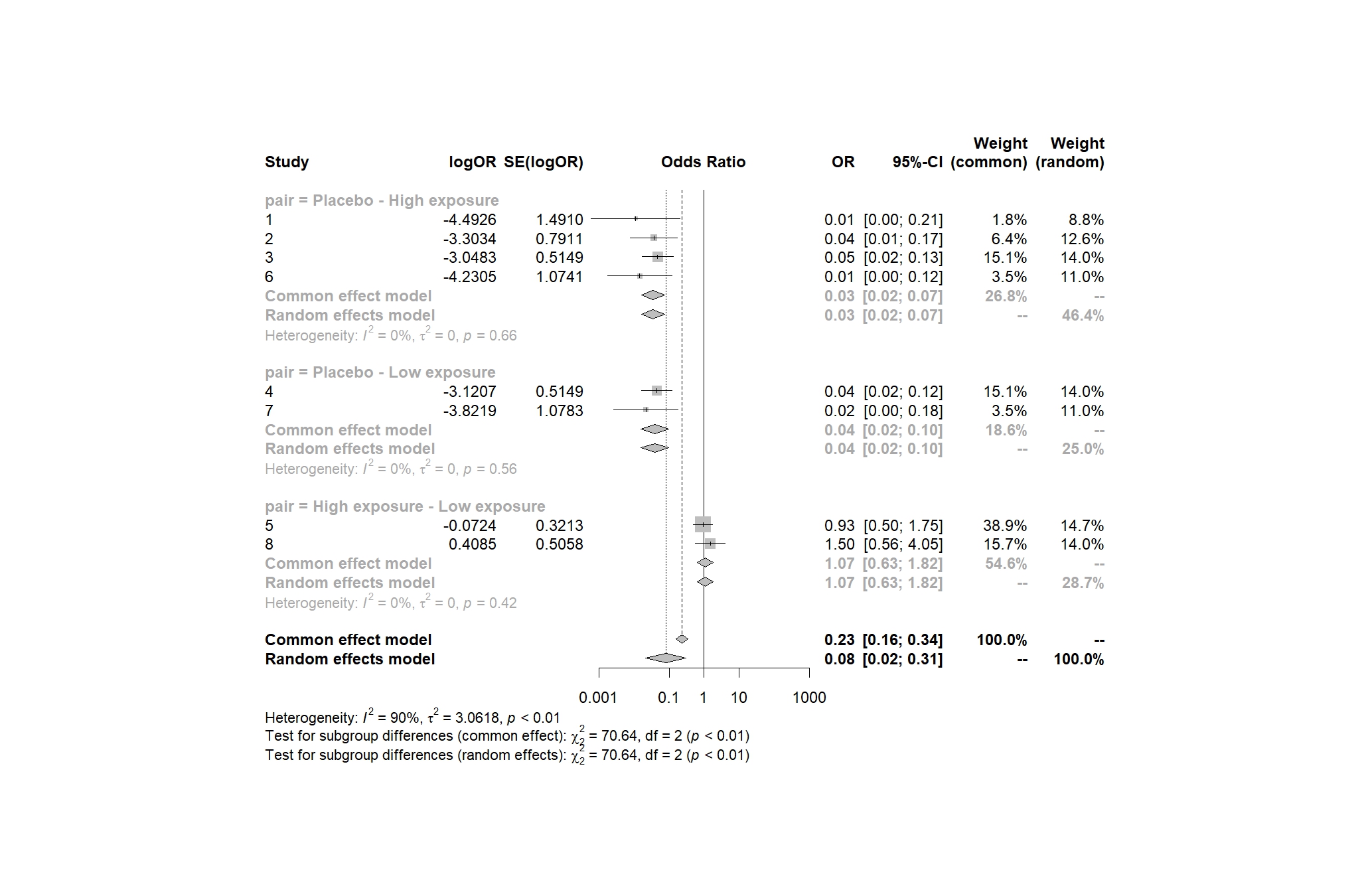

Supplement: Supplementary file 1 [file life-15-00307-s001.zip › Figure S2 new.jpeg]
